# Non-Lamellar Liquid Crystalline Nanocarriers for Thymoquinone Encapsulation

**DOI:** 10.3390/molecules25010016

**Published:** 2019-12-19

**Authors:** Anan Yaghmur, Boi Vi Tran, Seyed Moein Moghimi

**Affiliations:** 1 Department of Pharmacy, Faculty of Health and Medical Sciences, University of Copenhagen, Universitetsparken 2, DK-2100 Copenhagen Ø, Denmark; boivitran@msn.com; 2 School of Pharmacy, Newcastle University, Newcastle upon Tyne NE1 7RU, UK; moein.moghimi@gmail.com; 3 Translational and Clinical Research Institute, Newcastle University, Newcastle upon Tyne NE2 4HH, UK; 4 Colorado Center for Nanomedicine and Nanosafety, Skaggs School of Pharmacy and Pharmaceutical Sciences, University of Colorado Anschutz Medical Campus, Aurora, CO 80045, USA

**Keywords:** drug encapsulation, inverse cubic *Fd3m* phase, inverse hexagonal phase, nanodispersions, micellar cubosomes, nanoparticle tracking analysis, synchrotron small-angle scattering

## Abstract

Owing to their unique structural features, non-lamellar liquid crystalline nanoparticles comprising cubosomes and hexosomes are attracting increasing attention as versatile investigative drug carriers. Background: Depending on their physiochemical characteristics, drug molecules on entrapment can modulate and reorganize structural features of cubosomes and hexosomes. Therefore, it is important to assess the effect of guest molecules on broader biophysical characteristics of non-lamellar liquid crystalline nanoparticles, since drug-induced architectural, morphological, and size modifications can affect the biological performance of cubosomes and hexosomes. Methods: We report on alterations in morphological, structural, and size characteristics of nanodispersions composed from binary mixtures of glycerol monooleate and vitamin E on thymoquinone (a molecule with wide therapeutic potentials) loading. Results: Thymoquinone loading was associated with a slight increase in the mean hydrodynamic nanoparticle size and led to structural transitions from an internal biphasic feature of coexisting inverse cubic *Fd3m* and hexagonal (H_2_) phases to an internal inverse cubic *Fd3m* phase (micellar cubosomes) or an internal inverse micellar (L_2_) phase (emulsified microemulsions, EMEs). We further report on the presence of “flower-like” vesicular populations in both native and drug-loaded nanodispersions. Conclusions: These nanodispersions have the potential to accommodate thymoquinone and may be considered as promising platforms for the development of thymoquinone nanomedicines.

## 1. Introduction

*Nigella sativa*, commonly known as black cumin, fennel flower, and kalonji, is an annual herbaceous plant with a rich religious and historical background [1,2,3]. It belongs to the botanical Ranunculaceae family and is used in the preparation of herbal medicines [1,2,3,4,5,6,7]. It is native to North Africa, Southern Europe, and Southwest Asia, and is cultivated in India, Pakistan, and Middle Eastern and Mediterranean countries [7]. Black cumin seeds and its extracted oil have been indicated for potential treatment and prevention of a variety of conditions including bronchial asthma, eczema, infectious diseases (e.g., influenza, dysentery), obesity, hypertension, gastrointestinal disorders, and pain relief [1,2,3,4,5,6,7].

The therapeutic potentials of black cumin seeds are mainly attributed to thymoquinone (TQ), which is the main active compound (30–48%) of the volatile (essential) oil of *Nigella sativa* [1,2,3,4,7,8]. TQ, 2-isopropyl-5-methylbenzo-1,4-quinone, is a quinone with relatively poor water solubility (0.549–0.740 g/L) [8,9]. Among its pharmacological properties, TQ displays antioxidant, antihistamine, immunomodulatory, anti-inflammatory, anti-microbial, and neuroprotective activities [2,5,6,7,10,11]. It was also reported that TQ is a potent anticancer agent and has potential in the prevention and treatment of breast, colon, and ovarian cancers [4,9,10,12,13,14].

Despite its broad therapeutic potentials, there are major concerns and limitations (including low solubility in water, poor bioavailability, poor membrane penetration capacity, and lack of mechanistic understanding of the drug action) hindering the translational development of TQ-based formulations [4,5,9,10]. Overcoming these limitations might be achieved through the rational design of nanoparticulate formulations for TQ delivery [4,8,11,15,16,17,18]. Taking into account the current interest in the development of nanocarriers for delivering TQ and other phytochemicals [8,9,10,11,15,16,17,18,19,20,21], here we focus on non-lamellar liquid crystalline nanoparticles enveloping a biphasic feature of inverse discontinuous cubic (I_2_) phase of the symmetry *Fd3m* phase coexisting with an inverse hexagonal (H_2_) phase for TQ encapsulation. The unique structural properties of these nano-self-assemblies and their analogues [22,23,24,25,26,27,28,29,30,31,32,33,34,35,36,37,38,39,40,41] render them particularly attractive for enhancing the solubilization capacity of poorly water-soluble drugs and improving their delivery through different routes of administration [23,26,27,29,36]. However, the structural features of this family of liquid crystalline nanoparticles is highly sensitive to drug loading, and subsequently could affect its stability as well as biological performance. Here, we examine the effect of TQ loading on the structural and morphological features of non-lamellar liquid crystalline nanoparticles from a binary lipid mixture consisting of glycerol monooleate (GMO), which is the most investigated lipid with non-lamellar liquid crystalline phase forming propensity, and vitamin E (Vit E). These preparations were stabilized with d-α-tocopheryl poly(ethylene glycol)_2000_ succinate (TPGS-PEG2000) and their characteristics were investigated through a pan-integrated approach involving synchrotron small angle X-ray scattering (SAXS), nanoparticle tracking analysis (NTA), and cryo-transmission electron microscopy (Cryo-TEM). We further report on TQ encapsulation efficiency and the effect of ageing on the mean nanoparticles sizes within 30 days of post-preparation of structurally optimized samples.

## 2. Results and Discussion

### 2.1. Biophysical Characteristics of Drug-Free GMO/Vit E Nano-Self-Assemblies

**Figure 1:**
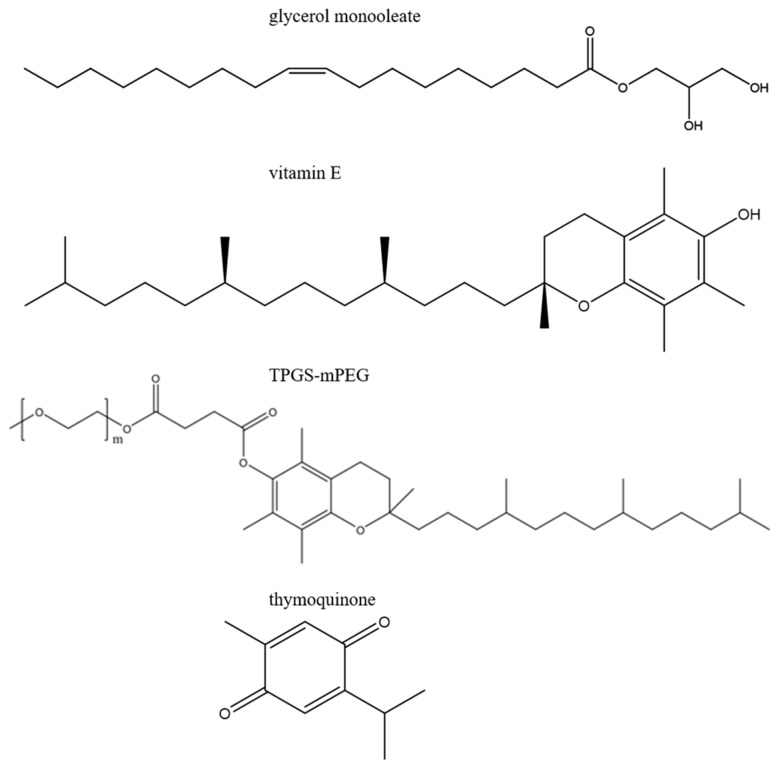
The molecular formulas of glycerol monooleate (GMO), vitamin E (Vit E), d-α-tocopheryl poly(ethylene glycol)_2000_ (TPGS-mPEG; m: 2000), and thymoquinone (TQ).

**Figure 2:**
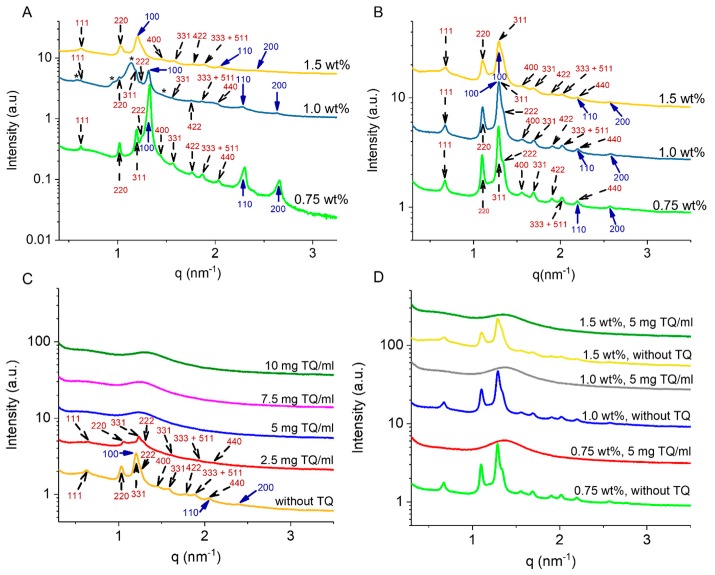
Effects of stabilizer (TPGS-PEG2000) and TQ loading on the internal nanostructure of GMO/Vit E nanoparticles. SAXS patterns at 37 °C for (**A**,**B**) TQ-free and (**C**,**D**) TQ-loaded GMO/Vit E nanodispersions contained a constant total lipid (GMO/Vit E binary mixture) concentration of 5.0 wt%. These samples were prepared at two different GMO/Vit E weight ratios: (**A**,**C**) 70:30 and (**B**,**D**) 60:40. In (**A**), (**B**), and (**D**), the samples were stabilized with TPGS-PEG2000 at three different concentrations of 0.75, 1.0, and 1.5 wt%; whereas those in panel (**C**) were stabilized using the same stabilizer at a constant concentration of 1.5 wt%. The Bragg peaks are represented with black dash arrows and blue solid arrows for the corresponding Miller indices of the cubic *Fd3m* and H_2_ phases, respectively. In (**A**), the detected characteristic Bragg peaks for the second coexisting cubic *Fd3m* phase at TPGS-PEG2000 content of 1.0 wt% are marked with an asterisk. In all panels, the intensities were shifted by a constant arbitrary factor for better visibility.

Figure 1 shows the molecular formulas of TQ, TPGS-PEG2000, and the lipid constituents of the nanodispersions. Composition and main biophysical characteristics of TQ-free and TQ-loaded nanodispersions are presented in Table 1 and Table 2. The interior organization of GMO/Vit E (weight ratios of 70:30 and 60:40) nano-self-assemblies stabilized with different concentrations of TPGS-PEG2000 (0.75–1.5 wt%) was studied by synchrotron SAXS. With both lipid compositions, the SAXS patterns for the TPGS-PEG2000-stabilized nanodispersions (Figure 2A,B) indicated the formation of species enveloping an internal biphasic feature of coexisting inverse hexagonal (H_2_) phase and inverse discontinuous (micellar) cubic phase of the symmetry *Fd3m*. The identification of these phases presented in Figure 2A,B was based on the detection of the first three characteristic peaks for the former phase (reflections marked with blue solid arrows: (100), (110), and (200)) and at least nine characteristic Bragg peaks (reflections marked with black dash arrows: (111), (220), (311), (222), (400), (331), (422), (511), (333), (440)) for the latter phase. The inverse cubic *Fd3m* (Q^227^) phase is a three-dimensional (3D) nanostructure enveloping quasi-spherical micelles with two different sizes and is the most identified micellar cubic phase in lipid systems [38,42,43,44,45,46,47], and previously observed between the inverse hexagonal (H_2_) and micellar (L_2_) phases in both dispersed and non-dispersed systems based on binary mixtures of unsaturated monoglyceride (e.g., GMO, monolinolein, and monoelaidin) and solubilized oil (e.g., Vit E, oleic acid, tetradecane, and elaidic acid) [38,44,45,46].

Notably, increasing TPGS-PEG2000 concentration at both lipid compositions, particularly at GMO/Vit E ratio of 60:40 (Figure 2B), did not have a significant influence on the lattice parameters of both coexisting H_2_ and cubic *Fd3m* phases (Table 1). This is most likely attributed to the tendency of TPGS-PEG2000 to form micelles and vesicles as previously reported [48,49]. At GMO/Vit E ratio of 70:30 (Figure 2A), the results showed a slight increase in the lattice parameter of the coexisting H_2_ phase on increasing TPGS-PEG2000 concentration from 0.75 to 1.5 wt%. Furthermore, the SAXS pattern showed traces of an additional cubic *Fd3m* phase with Bragg peaks (marked with asterisks) having a lattice parameter of approximately 18.60 nm at 1.0 wt% TPGS-PEG2000 (Figure 2A, dark blue SAXS pattern). These structural alterations are most likely attributed to the accommodation of relatively small amounts of TPGS-PEG2000 in the internal nanostructure of GMO/Vit E nanoparticles leading to a slight enlargement of hydrophilic domains. These observations are in line with previous studies showing that the incorporation of PEGylated stabilizers into hydrophilic domains of lipid nanodispersions can induce significant structural alterations and phase transitions [24,25,50,51]. For instance, we recently reported on the significant influence of three different poly(ethylene glycol)-grafted 1,2-distearoyl-*sn*-glycero-3-phosphoethanolamines (DSPE-mPEG), namely DSPE-PEG350, DSPE-PEG750, and DSPE-PEG2000, on the enlargement of internal hydrophilic water channels in bicontinuous cubic *Pn3m* and *Im3m* phases of cubosomes based on phytantriol [51].

**Figure 3:**
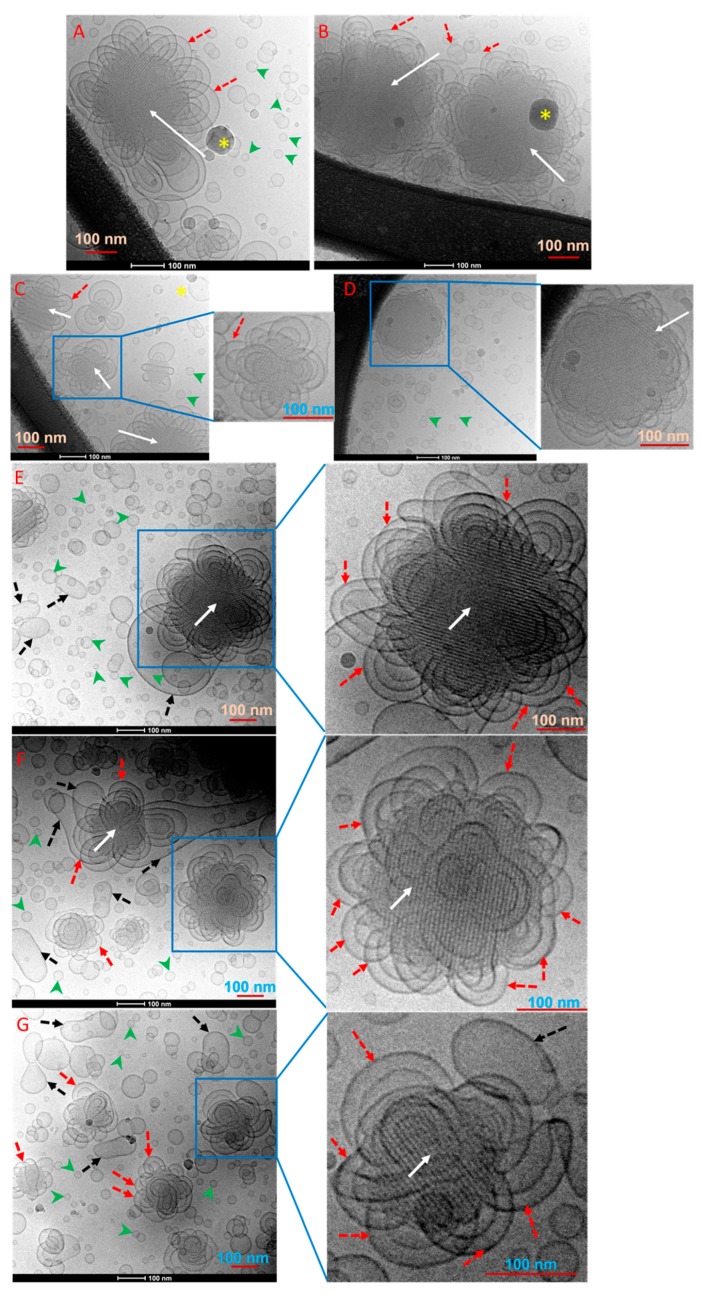
Cryo-transmission electron microscopy (TEM) images of (**A**–**D**) TQ-free and (**E**–**G**) TQ-loaded samples prepared at GMO/Vit E weight ratio of 70:30 and stabilized with TPGS-PEG2000 at a concentration of 1.5 wt% (samples 3 and 7, respectively in Table 1). These GMO/Vit E nanodispersions were prepared at a total lipid concentration of 5.0 wt% and contained TQ at concentrations of 0 (sample 3) and 2.50 mg/mL (sample 7), respectively. In these images, the visible non-lamellar liquid crystalline nanoparticles are marked with solid white arrows, whereas the adsorbed flower-like vesicular structures on the nanoparticles’ surfaces are marked with red dash arrows. In (**A**), (**B**), and (**D**), the dark spot indicating the formation of an ice crystal on the microscope grid is marked with a yellow asterisk. In all panels, the coexisting small nano-objects (most likely vesicles) are marked with green arrowheads; whereas the visible larger deformed and elongated vesicular structures are marked with solid dash arrows. In all images and enlarged areas, the scale bar is 100 nm.

Depending on the lipid composition, the stabilizer type, and its concentration, non-lamellar liquid crystalline nanodispersions display a typically heterogeneous environment of coexisting morphologies [24,25,32,52,53,54,55,56]. For instance, Pluronic F127-stabilized cubosomes and hexosomes typically coexist with vesicles [22,54,57,58]. Accordingly, we used cryo-TEM to gain further insight into the morphological features of the non-lamellar liquid crystalline nanoparticles and their possible coexistence with vesicles, micelles, or other species. For a selected nanodispersion prepared at GMO/Vit E weight ratio of 70:30 and TPGS-PEG2000 concentration of 1.5 wt%, the recorded cryo-TEM images displayed species with diameters in the range of a few tens to a few hundred nanometers (Figure 3A–D). Furthermore, the images show the presence of “flower-like” vesicles (marked with red dash arrows) covering the non-lamellar liquid crystalline nanoparticles (marked with white solid arrows) that have sizes in the range of 130–270 nm. These morphologies were not observed in cubosomes, hexosomes, and other related nano-self-assemblies stabilized with Pluronic F127 [45,52,53,54,57,59]. Based on SAXS findings (Figure 2A), these visible non-lamellar liquid crystalline nanoparticles are most likely micellar cubosomes with an internal cubic *Fd3m* phase, since there was no indication (Figure 3) on the formation of hexosomes with their typical morphological features of hexagons and curved striations [60].

NTA was used to gain insight into the hydrodynamic size and size distribution of selected GMO/Vit E nanodispersions. NTA results showed that the mean hydrodynamic diameters and modes for the two nanodispersions prepared at GMO/Vit E weight ratio of 60:40 (control samples 4 and 6, Table 2) were increased from 119 ± 49 and 95 ± 3 nm to 141 ± 53 and 131 ± 3 nm, respectively on increasing TPGS-PEG2000 concentration from 0.75 to 1.5 wt%. The reason for such a size increase is unclear, but this may be attributed to the occurrence of a higher fraction of coexisting relatively large vesicles with the non-lamellar liquid crystalline nanoparticles on increasing TPGS-PEG2000 concentration. At TPGS-PEG2000 concentration of 1.5 wt%, an increase in GMO/Vit E weight ratio to 70:30 (sample 1, Table 2) did not induce a significant change in the mean nanoparticle size and mode.

### 2.2. Effect of TQ Loading on Biophysical Characteristics of GMO/Vit E Nano-Self-Assemblies

We investigated the effect of TQ loading on biophysical characteristics of selected TPGS-PEG2000-stabilized GMO/Vit E nanodispersions (Table 1, Figure 2C,D). Loading TQ at 2.5 mg/mL led to a structural transition from an internal biphasic cubic *Fd3m*/H_2_ feature (vivid orange SAXS pattern, Figure 2C) having lattice parameters of 17.34 and 6.02 nm, respectively, to micellar cubosomes with an internal neat cubic *Fd3m* phase having a lattice parameter of 16.81 nm (red SAXS pattern, Figure 2C). On further increasing TQ concentration (5–10 mg/mL), a structural transition from an internal cubic *Fd3m* phase (micellar cubosomes) to an internal L_2_ phase (formation of emulsified microemulsions, EMEs) was observed. The latter phase was characterized by the appearance of a single broad peak with corresponding *q* values of 1.27, 1.27, and 1.34 nm^−1^, respectively, with increasing TQ concentration from 5 to 7.5, and 10 mg/mL (Figure 2C). This change in *q* was associated with a slight decrease in the characteristic distance of the internal L_2_ phase from 4.95 to 4.69 nm (Table 1). At GMO/Vit E ratio of 60:40 and the following three different concentrations of TPGS-PEG2000: 0.75, 1.0, and 1.5 wt% (Table 1 and Figure 2D), a similar phase transition was detected from an internal biphasic cubic *Fd3m*/H_2_ feature to an internal L_2_ phase on loading TQ at a constant concentration of 5 mg/mL. It was evident that TQ has a significant effect on the internal nanostructure of GMO/Vit E nano-self-assemblies, with almost no effect of the stabilizer in the investigated concentration range as the characteristic distance of the internal L_2_ phase was similar at the three employed TPGS-PEG2000 concentrations. This observation might be attributed to preferential formation of coexisting micelles and vesicles on increasing TPGS-PEG2000 concentration as discussed above. These detected cubic *Fd3m*/H_2_-L_2_ phase transitions on increasing TQ concentration are consistent with previous reports [22,31,43,45,54,61,62,63,64] on the influence of solubilizing a hydrophobic additive such as medium chain triglycerides and oleic acid on the structural features of non-lamellar liquid crystalline phases (in both dispersed and bulk (non-dispersed) states). It was reported that the localization of such guest molecules in the hydrophobic (lipidic) domains of the internal non-lamellar liquid crystalline phase leads in a concentration-dependent manner to the following typical colloidal transformations: cubosomes with an internal bicontinuous cubic phase, via hexosomes with an internal H_2_ phase, to micellar cubosomes and EMEs with internal cubic *Fd3m* and L_2_ phases, respectively [43,45,54,64]. A number of plausible reasons might account for this sequential phase transition in the nanoparticles’ interiors [42,43,44,54,65], such as associated change with water quantity (dehydration effect), increase in the effective volume of the acyl chains of the main surfactant-like lipid constituents, and release of packing frustrations by filling out the interstitial regions of the hydrophobic domains of the internal nanostructure.

Cryo-TEM was further employed to shed light on the effect of loading TQ on morphological characteristics of selected nanodispersions. In Figure 3E–G, the cryo-TEM images from TQ encapsulated (2.5 mg/mL) GMO/Vit E nanodispersion (weight ratio 70:30; 1.5 wt% TPGS-PEG2000) displayed morphologies with size diameters in the range of a few tens to a few hundred nanometers and similar to those detected in the corresponding TQ-free nanodispersion (Figure 3A–D). The nature of the cubic *Fd3m* phase [52,66], however, creates a technical challenge in visualizing the internal structure of TQ-free and TQ-loaded nanodispersions by cryo-TEM (Figure 3), since this phase is composed of two different closely packed quasi-spherical reversed micelles with relatively low amounts of “solubilized” water, and a reduced contrast between the lipid and water domains compared with nanoparticles with internal H_2_ and V_2_ phases in hexosomes and cubosomes, respectively that generally have larger water domains [45,52,54,66]. The images further show the presence of coexisting small spherical morphologies (marked with green arrowheads) in a size range of 15–55 nm (Figure 3). These small aggregates are most likely representing coexisting vesicles. Considering the tendency of TPGS-PEG2000 to form normal micelles (L_1_ phase) [48,49], we do not exclude the coexistence of micelles in these nanodispersions. In addition to these, other relatively larger deformed and elongated vesicular structures (marked with black dash arrows) were also visible in cryo-TEM images (Figure 3).

For nanodispersions prepared by employing the same TPGS-PEG2000 concentration, it was evident from NTA findings that loading TQ at a concentration of 5 mg/mL was associated with a slight increase in the mean nanoparticle hydrodynamic size and mode (Table 2). This is most likely attributed to the observed TQ-mediated structural changes in GMO/Vit E nano-self-assemblies (Figure 2C). Previous investigations have also confirmed an increase in the mean nanoparticle size on TQ encapsulation in other lipidic and polymeric-based nanocarriers [11,67].

**Figure 4:**
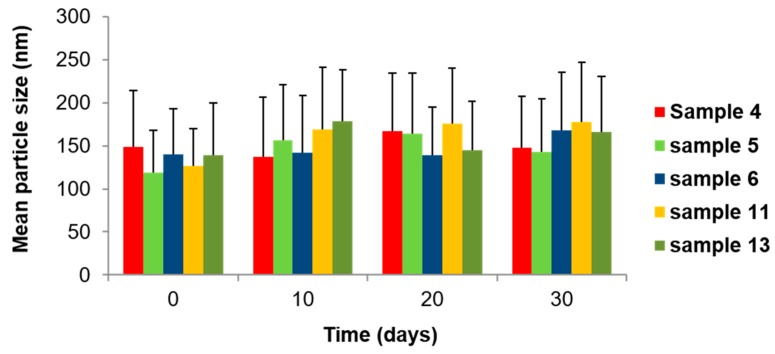
Effect of ageing on mean hydrodynamic nanoparticle size for TQ-free and TQ-loaded GMO/Vit E nanodispersions. All nanodispersions were prepared at a constant GMO/Vit E ratio of 60:40 and total lipid (GMO plus Vit E) concentration of 5.0 wt%. The TQ-free (control) samples were stabilized with TPGS-PEG2000 at the following concentrations: 0.75 wt% (sample 4, red bars), 1.0 wt% (sample 5, green bars), 1.5 wt% (sample 6, dark blue bars). The two selected TQ-loaded samples were prepared at TQ concentration of 5 mg/mL and stabilized by TPGS-PEG2000 at 0.75 wt% (sample 11, orange bars) and 1.5 wt% (sample 13, olive green bars). The samples were analyzed at room temperature at the following time points: 0 (preparation day), 10, 20, and 30 days.

Finally, we also assessed the effect of ageing on the nanoparticle size at ambient temperature within a month of preparation. The results demonstrated no significant changes in the mean nanoparticle size, regardless of lipid composition, stabilizer or drug concentration (Figure 4). GMO/Vit E nanodispersions were also visually inspected and found to be colloidally stable for at least three months after preparation.

### 2.3. Encapsulation Efficiency of TQ-Loaded GMO/Vit E Nano-Self-Assemblies

The encapsulation efficiency (EE) of TQ was tested on three selected GMO/Vit E nanodispersions prepared at GMO/Vit E weight ratios of 70:30 and 60:40 and found to be 99.0 wt% in the preparation day (Table 2). This observation was consistent with previous reports on TQ encapsulation in Tween 80-stabilized lipid carriers based on hydrogenated palm oil, lecithin, and olive oil, and liposomes based on 1,2-dipalmitoyl-*sn*-glycero-3-phosphocholine (DPPC) [11,17]. In line with previous investigations on the EE of poorly water-soluble drugs [11,17,68], the obtained high EE in this study is most likely attributed to high lipophilicity of TQ and its preferential localization within the hydrophobic domains of the internal nanostructures of these GMO/Vit E nano-self-assemblies. The EE of TQ was also determined for two selected samples at GMO/Vit E weight ratio of 60:40 (samples 11 and 13 in Table 2) after five days of preparation, and the results indicated a marginal decrease in the EE (Table 2).

## 3. Materials and Methods

### 3.1. Materials

Glycerol monooleate (GMO, product name: RIKEMAL-XO-100) with a purity ≥ 90% was obtained from Riken Vitamin Co. (Tokyo, Japan). Vitamin E (Vit E) with a purity ≥ 96% was purchased from Sigma Aldrich (Helsinki, Finland). d-α-tocopheryl poly (ethylene glycol)_2000_ succinate (TPGS-PEG2000) was purchased from Isochem (Vert-Le-Petit, France). Thymoquinone (TQ, purity ≥ 99%) was purchased from Sarchem Laboratories, Inc. (Farmingdale, NJ, USA). Phosphate buffered saline (PBS) tablets at pH 7.4 were purchased from Sigma Aldrich (Poole, UK). For preparing the 0.01 M PBS buffer, Milli-Q water was collected from Millipore Direct-Q3 UV system (Billerica, MA, USA). Ethanol with a purity ≥96% was obtained from Merck (Darmstadt, Germany). All ingredients were used without further purification.

### 3.2. Preparation of Thymoquinone-Free and Thymoquinone-Loaded GMO/Vit E Nanodispersions

The TQ-free and TQ-loaded nanodispersions were prepared using binary GMO/Vit E mixtures at the following four GMO/Vit E weight ratios: 90:10, 70:30, 60:40, and 50:50. These nanodispersions were prepared at different TQ concentrations ranging from 0 to 7.5 mg/mL, and the total lipid (binary GMO/Vit E mixture) concentration was kept constant at 5.0 wt%. GMO was first melted at ~40 °C and weighed into a glass vial followed by the addition of Vit E and vortexing to obtain a clear lipid binary solution. To prepare TQ-loaded nanodispersions, appropriate amounts of TQ were dissolved in the molten binary lipid GMO/Vit E mixtures by a gentle vortexing. Preheated TPGS-PEG2000 at concentrations in the range of 0.75–1.5 wt% and 60 °C were then added, and the obtained clear lipid solutions were gently vortexed in excess of 0.01 M phosphate buffer (pH 7.4). These samples were then subjected to ultrasonication by using the ultrasonic processor Qsonica 500 (Qsonica LLC, Newtown, CT, USA) for 5 min in pulse mode (5 s pulses interrupted by 2 s breaks) at 25% of its maximum power (500 W). Based on visual inspections, these produced GMO/Vit E nanodispersions were found to be colloidally stable for at least three months of post-preparation at room temperature.

### 3.3. Size Characterization of GMO/Vit E Nanodispersions

Size characterization of the nanoparticles in six selected TQ-free and TQ-loaded GMO/Vit E nanodispersions was conducted as recently described in [29] by using NanoSight NS300 mounted with a 405 nm laser and a microscope (Malvern Panalytical Ltd., Worcestershire, UK). Briefly, the size measurements were performed in triplicate at room temperature, and the data represent the obtained average values of these runs. Prior to measurements, the samples were diluted in filtered PBS buffer (pH 7.4) to reach nanoparticle concentration between 10^8^ and 10^9^ nanoparticles/mL. In these measurements, the recorded videos were analyzed using Malvern software (NTA 3.2 Dev Build 3.2.16, Malvern Panalytical Ltd., Worcestershire, UK).

### 3.4. Cryo-Transmission Electron Microscopy (Cryo-TEM)

The morphological characterization of the selected TQ-free and TQ-loaded nanodispersions was performed, as described in our recent reports [34,35]. Briefly, 3–4 µL of the nanodispersion was applied on a hydrophilized lacey carbon 300 mesh copper grid (Ted Pella Inc., Redding, CA, USA). The observations were done with Tecnai G2 20 transmission electron microscope (FEI, Eindhoven, Holland) at a voltage of 200 kV under a low-dose rate (~5 e/Å^2^s). Images were then recorded using a FEI Eagle camera 4k × 4k at a nominal magnification of 69,000× resulting in a final image sampling of 0.22 nm/pixel.

### 3.5. Synchrotron Small-Angle X-ray Scattering (SAXS)

The experiments were performed on the I22 beamline at the Diamond Light Source synchrotron at Harwell Campus (Didcot, UK). An X-ray beam having a wavelength of 0.9998 Å at X-ray energy of 12.4 KeV was used, with a sample-to-detector distance of 3563.3 mm, and a beam size of 120 × 370 μm^2^. The 2D SAXS patterns were acquired using a Pilatus 2M from Dectris Ltd. (Baden, Switzerland). Silver behenate (CH_3_-(CH_2_)_20_-COOAg with a *d*-spacing value of 58.38 Å) was used as a standard to calibrate the angular scale of the measured intensity. In our investigations, 1 mm diameter quartz capillaries (sample holders) were used and the measurements were performed by sealing the samples in the thin-walled glass capillaries at 37 °C with an exposure time of 5 s per frame with 2 s delay between 3 frames. The *q* range was 0.10 to 6.0 nm^−1^ (*q = 4π sin θ/λ*, where *λ* is the wavelength and *2θ* is the scattering angle). The software, Dawn version 2.7, available with 2D data reduction pipeline perspective was used for SAXS data reduction. After subtracting the background scattering using PBS buffer, all Bragg peaks were fitted by Lorentzian distributions. The lattice parameters of the internal inverse discontinuous cubic *Fd3m* and hexagonal (H_2_) phases of GMO/Vit E nanoparticles were derived from the SAXS diffraction patterns. We note that in each respective phase regime, only the strongest reflections were considered for calculating the corresponding lattice parameters. The scattering profile of the inverse micellar solution (the L_2_ phase) was characterized by the appearance of a single broad peak, which was fitted for calculating its characteristic distance, *d = 2π/q*.

### 3.6. Encapsulation Efficiency

The encapsulation efficiency (EE) of TQ was determined by using the following indirect method: the free (un-encapsulated) TQ was isolated from the nanodispersion by centrifugation (9600 rcf) for 10 min using a microcentrifuge (VWR^TM^ 1207 Galaxy 7D^®^ Centrifuge, Radnor, PA, USA) and then its concentration was determined, as described in the previous report of Salmani et al. [69], at a wavelength of 257 nm by using Cary 60 UV-VIS spectrophotometer (Agilent Technologies, Santa Clara, CA, USA). For TQ quantification, a calibration curve was prepared by dissolving the drug in ethanol at different concentrations in the range of 0.5–10 μg/mL.

TQ encapsulation efficiency (EE) was calculated using the following equation [36]:

(1) $$EE\;\left(\%\right)=\frac{C_{total\;}-C_{free}}{C_{total}}\times 100\%$$

where *C_free_* refers to the concentration of un-encapsulated TQ as determined by UV-VIS spectrophotometer; whereas *C_total_* is the initial concentration of TQ in the nanodisperison.

## 4. Concluding Remarks

Non-lamellar liquid crystalline nanoparticles are attracting increasing interest for drug delivery applications. For evaluating the potential pharmaceutical use of these nano-self-assemblies, it is important to gain insight into the influence of lipid composition and drug loading on their structural features, size, and morphological characteristics. The present study demonstrated that TQ in a concentration-dependent manner dramatically alters the internal nanostructure as well as the morphological features of TPGS-PEG2000-stabilized GMO/Vit E nanoparticles. Furthermore, cryo-TEM observations demonstrated that native as well as TQ-loaded GMO/Vit E nanodispersions coexist with vesicles, relatively small spherical nano-objects, and vesicular nanostructures with “flower-like” features. More specifically, the ‘lamellar petals’ morphologies were covering the outer surfaces of nanoparticles with a dense core of non-lamellar liquid crystalline structures.

Taking into account the important role of nanoparticle shape and surface characteristics on modulating interactions with biological fluids, enzymes (e.g., lipases), and cells [70,71,72,73], future studies are necessary to assess the performance of TQ-loaded non-lamellar liquid crystalline nanoparticles in appropriate biological environments and animal models, and evaluate their drug release properties. This could lead to design optimization and formulation validation with necessary regulatory attributes for reproducible pharmaceutical development.

## Figures and Tables

**Table 1:** Composition and biophysical characteristics of engineered drug-free and TQ-loaded TPGS-PEG2000-stabilized GMO/Vit E nanodispersions. Synchrotron small angle X-ray scattering (SAXS) experiments were performed at 37 °C.

Sample	Lipids	Stabilizer (wt%)	TQ (mg/mL)	Space Group	Lattice Parameter (nm)
	(GMO/Vit E Ratio)				
1	70:30	0.75	0	*Fd3m* H_2_	17.45 5.45
2	70:30	1.0	0	*Fd3m* *Fd3m^a^* H_2_	17.45 18.60 5.50
3	70:30	1.5	0	*Fd3m* H_2_	17.34 6.02
4	60:40	0.75	-	*Fd3m* H_2_	16.20 5.64
5	60:40	1.0	-	*Fd3m* H_2_	16.20 5.64
6	60:40	1.5	-	*Fd3m* H_2_	16.20 5.64
7	70:30	1.5	2.5	*Fd3m*	16.81
8	70:30	1.5	5	L_2_^b^	4.95
9	70:30	1.5	7.5	L_2_^b^	4.95
10	70:30	1.5	10	L_2_^b^	4.69
11	60:40	0.75	5	L_2_^b^	4.55
12	60:40	1.0	5	L_2_^b^	4.53
13	60:40	1.5	5	L_2_^b^	4.53

^a^ The lattice parameter of the coexisting second cubic *Fd3m* phase that was identified based on the detection of four weak reflections (marked with asterisk in Figure 2A). ^b^ The characteristic distance of the inverse micellar (L_2_) phase.

**Table 2:** Hydrodynamic size characteristics and encapsulation efficiency (EE) of selected TQ-free and TQ-loaded GMO/Vit E) nanodispersions ^a^.

Sample	Lipids	Stabilizer (wt%)	TQ (mg/mL)	Mean Particle Size ± SD (nm)	Mode ± SD (nm)	EE ± SD (%)	EE ± SD (%)
	(GMO/Vit E Ratio)					Preparation Day	5 Days
1	70:30	1.5	0	145 ± 49	126 ± 11		
4	60:40	0.75	0	119 ± 49	95 ± 3		
6	60:40	1.5	0	141 ± 53	131 ± 3		
8	70:30	1.5	5	160 ± 65	134 ± 7	-	-
11	60:40	0.75	5	127 ± 43	118 ± 3	98.8 ± 0.09	97.6 ± 0.11
13	60:40	1.5	5	168 ± 67	136 ± 1	98.9 ± 0.10	97.9 ± 0.09
14	70:30	1.5	7.5	-	-	99.4 ± 0.05	-

^a^ Nanoparticle tracking analysis (NTA) measurements were conducted on selected TQ-loaded nanodispersions prepared at TQ concentration of 5 mg/mL. The EE of TQ in the selected nanodispersions was determined in the preparation day (day 0) and after 5 days of preparation.
